# Age-Related Cognitive Decline May Be Moderated by Frequency of Specific Food Products Consumption

**DOI:** 10.3390/nu13082504

**Published:** 2021-07-22

**Authors:** Aleksandra Bramorska, Wanda Zarzycka, Wiktoria Podolecka, Katarzyna Kuc, Aneta Brzezicka

**Affiliations:** 1 SWPS Institute of Psychology, University of Social Sciences and Humanities, 03-815 Warsaw, Poland; wzarzycka@swps.edu.pl (W.Z.); wpodolecka@st.swps.edu.pl (W.P.); kkuc@swps.edu.pl (K.K.); abrzezi2@swps.edu.pl (A.B.); 2 Faculty of Information Technology, Polish-Japanese Academy of Information Technology, 02-008 Warsaw, Poland

**Keywords:** Western diet, healthy diet, food consumption, cognitive functioning, cognitive aging, memory, arithmetic abilities, the SynWin multitasking task, Fatigue Assessment Scale (FAS)

## Abstract

Our study aimed to evaluate whether the type of food products and the frequency of their consumption are associated with cognitive functioning in younger and older adults. The impact of diets that are high in added sugars and saturated fat on cognitive functioning, especially on memory, was at the center of our interest. Participants in the study were 204 healthy adults (aged 20–55) who performed a multitasking cognitive test and completed dietary and psychological questionnaires. Stepwise regression analysis with age and food consumption patterns as predictors, and the cognitive task performance as a dependent variable, revealed that cognitive task performance worsened with age. However, we found that the frequency of consuming different types of foods (healthy versus unhealthy dietary patterns) moderates the effects of age on cognitive functioning. Red meat and animal fat consumption were negatively correlated with cognitive performance, and this relation was dependent on the age of our participants. Conversely, white meat and fish consumption were positively related to memory. Different indices of dietary patterns (both positive and negative) were stronger predictors of cognitive performance in the older adult group. We interpret our results as evidence that diet may be a protective (or worsening) factor in age-related cognitive decline.

## 1. Introduction

Age-related cognitive decline is associated with the deterioration of many cognitive dimensions, including memory, cognitive control, attention, and working memory [1,2,3]. Diet is an important factor that may counteract age-related cognitive decline [4]. Further, research has shown that the hippocampus, a brain structure associated with a wide range of cognitive functions, may be influenced directly by diet or by diseases related to diet and eating habits, such as obesity or diabetes [5,6].

Diet is an integral part of preventing civilization diseases (e.g., obesity, diabetes, cardiovascular diseases) and medical treatment [7,8], and an important factor for mental health and cognitive performance [9,10,11]. The Mediterranean diet (MD) is considered to be one of the best dietary models for healthy aging and has been shown to decrease risk factors for cardiovascular diseases and dementia, for example [11]. The MD is a dietary pattern rich in antioxidants and, as such, has been suggested to have a protective effect on cognitive decline and dementia risk [11,12]. Fish is one of the key components of the MD and fatty fish is a good source of omega-3 polyunsaturated fatty acids (PUFAs), which are neuroprotective. Indeed, a recent large observational study reported that adherence to the MD was associated with a decreased risk of cognitive impairment and higher fish consumption was also associated with slower cognitive decline within the context of the MD [13]. Omega-3 PUFAs may also benefit memory performance by improving functional hippocampal connectivity [14].

In contrast, diets high in saturated fat, sugar, and animal protein and low in fiber, known as the Western diet (WS diet), have negative effects on health [4,15]. The WS diet lacks important polyphenols and anti-oxidant, may contain too little beneficial omega-3 PUFAs [5,8], and may cause mood and cognitive functioning decline [16] through hippocampus-dependent memory and learning deterioration [6,17,18]. It also leads to higher consumption of high-energy foods (especially high in saturated fat) and worsens hippocampus functioning, which may result in weight gain through behavioral changes [19,20].

Moreover, hippocampal-dependent memory deteriorates throughout the lifespan [21,22]. Attuquayefio et al. (2016) reported that being on a WS diet for only four days was sufficient to cause higher blood glucose levels, which resulted in worse memory task performance [23]. Glucose in circulating blood can facilitate cognitive functioning, especially by enhancing memory performance [24], but the quality and quantity of consumed products that are converted into glucose in the body are an important factor [25]. Lastly, the WS diet may cause blood–brain–barrier disruption and hippocampal-dependent memory deterioration [6,26].

Lipids are crucial for the development of the central nervous system [27] and PUFA is an important factor for maintaining brain functioning, e.g., synaptic plasticity or regulation of neurotransmission [28]. Horman et al. (2020) showed that the consumption of diets high in omega-6 PUFA, but with lower omega-3 PUFA, and conversely, changed the PUFA composition in brain structures, such as the hypothalamus, hippocampus, and prefrontal cortex [29]. Another study showed that even a short-term high-fat diet may increase the levels of corticosterone in the hippocampus and impair memory consolidation in rats [30]. The hippocampus is a structure responsible not only for memory and learning but also for controlling digestive functioning through hunger and satiety regulation [17]. The WS diet impairs hippocampal functioning and leads to a deficit in memory performance [31,32], resulting in weakened inhibitory control that leads to greater consumption of high-fat foods, further impairment, and weight gain [20,33].

The aim of the present research was to evaluate whether the type of food products and the frequency of their consumption are associated with cognitive functioning and whether they can moderate age-related changes in cognition. Age is an important factor in the deterioration process of a wide range of cognitive functions, and changes in the brain can alter cognitive functioning as early as just after the age of 35 [34]. We recruited participants in a wide age range to study the relation between diet and cognitive performance in various age groups.

Our main hypothesis was that high consumption of WS diet food products would result in a decrease in the performance on cognitive tasks, especially in the memory aspect. However, as cognitive functioning worsens with age we have also assumed that there are interaction effects of age and food patterns, with healthy foods diminishing the negative influence of age and unhealthy foods magnifying it.

## 2. Materials and Methods

### 2.1. Ethics

This study was accepted by the SWPS University of Social Sciences and Humanities Ethics Committee (no 41/2019), and all participants gave their informed consent prior to participation. Informed consent was given in accordance with the Declaration of Helsinki.

### 2.2. Participants

Participants were recruited by the Ariadna Nationwide Research Panel (NRP) in Poland. Volunteers were given points for participation in the study, which they could exchange for rewards.

All participants met the following qualifications for inclusion in the study: (1) age between 20 and 55 years old, (2) normal or corrected-to-normal visual acuity, (3) normal hearing, (4) no history of neurological or psychiatric disorders, (5) no injuries, including no previous head trauma, (6) no previous head or neck surgery and no brain tumors, (7) no use of medications known to affect cognition.

### 2.3. Data Collection

Data collection took place over the 12 consecutive weeks from September to October 2019. The study was conducted on-line and was divided into two 30 min long parts. The second part was completed within a week of finishing the first part. The first part included a personal questionnaire and one cognitive task (SynWin). The second part contained two dietary questionnaires, the Food Frequency Questionnaire (FFQ questionnaire), the Dietary Fat and free Sugar Short Questionnaire (DFS) [35], and the Fatigue Assessment Scale (FAS) and the NEO-Five Factor Inventory (NEO-FFI) personality scale [36]. However, the NEO-FFI and DFS questionnaires were not included in the analyses presented here.

### 2.4. Personal Data Assessment

#### 2.4.1. Personal Questionnaire

The personal questionnaire contained 35 questions about daily life and activities, personal information, and the participant’s state of health for the previous seven days. We included age (continuous), employment (nominal level), education (nominal level), health (1–10 range scale, where a higher value meant worse health (subjectively)), and the weight and height of our participants, which we then used to calculate the BMI index (body mass divided by the square of body height) in our analysis.

#### 2.4.2. Fatigue Assessment Scale

The Fatigue Assessment Scale (FAS) [37] was used to measure fatigue. The scale consisted of ten items in a 1–5 range scale addressing physical and mental fatigue. Two items were reverse-worded. The total score had a 10–50 range scale. Scores below 22 (≤21) indicated no fatigue, while a score of 22 and above indicated fatigue.

### 2.5. Dietary Assessment

#### Food Frequency Questionnaire

The Food Frequency Questionnaire (FFQ) [38] was used to obtain indicators of the quality and organization of the participant’s diet within the last year. The Food Frequency Questionnaire (FFQ) consisted of 111 items and was used to properly assess lifestyle, diet, and specific eating habits based on certain products. We included dietary knowledge (summary of correct answers about food and nutrition), pro-healthy and non-healthy diet indexes (described below in detail), diet type (omnivore, vegetarian), smoking (yes, no), sleep quality (7–8 h or less than 7/more than 8), physical activity (sedentary or light activity, active or moderately active, vigorously active) in our analysis.

The FFQ enabled the assessment of food consumed during the year and contained questions about the frequency and quantity of 165 food products. It included two assessment indicators of diet quality: (1) pro-healthy, consisting of 10 food groups with a beneficial effect on health, and (2) non-healthy, which was calculated from 14 items containing groups of food products unbeneficial to health. Both indexes were calculated according to the FFQ manual guide. The scores were calculated by summing up the frequency of particular food consumption and ranged from 0 to 100. The higher the index of the diet value was, the greater the concentration of features beneficial or unbeneficial to human health.

Indexes of pro-healthy and non-healthy dietary pattern values were both in the lower tertile. Furthermore, there was not much difference in the diet types of our participants. Because of these issues, we decided to analyze the frequency of consumption patterns of pro-healthy and non-healthy food products to check their dependence on each other and their influence on human cognitive functioning separately. Frequency consumption patterns were determined by using exploratory factor analysis separately for 10 pro-healthy group products and 14 non-healthy group products. Before performing the exploratory factor analysis the data were scaled to the 0–1 range, with 0 meaning consumption of the product less than once a month and 1 meaning consumption of more than once a day [39]. The transformation was carried out to facilitate the interpretation of the amount of consumed products. The Food Frequency Questionnaire (FFQ) was validated for the Polish population [40].

### 2.6. Cognitive Functioning Assessment–SynWin Task

**Figure 1:**
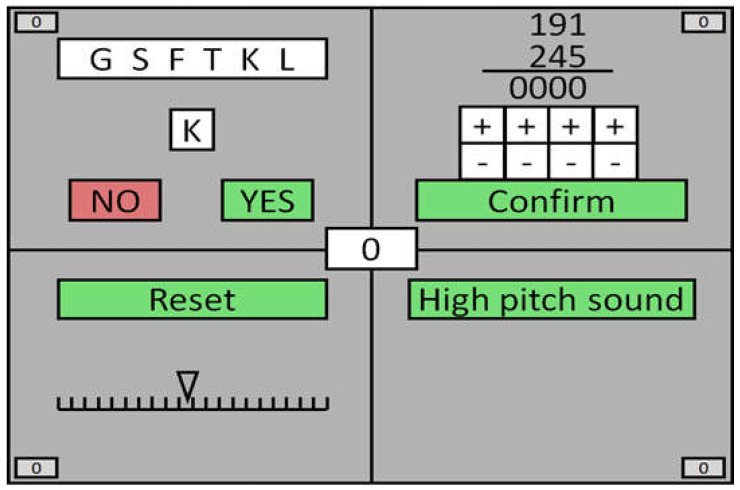
SynWin tasks (multitasking components): memory searching (upper left corner); arithmetic task (upper right corner); visual monitoring (lower left corner); auditory monitoring (lower right corner).

The SynWin test was used to measure multitasking abilities, with each subtask focusing on a different cognitive function: memory task, arithmetic task, visual monitoring, and auditory monitoring [41]. All components are presented simultaneously (Figure 1). The SynWin task application is divided into a practice block and three, 300 s main blocks. Participants receive or lose points depending on their actions during the SynWin task. Each component is described in detail below.

In memory searching, a set of six randomly chosen letters is presented for 5 s before disappearing abruptly. Then, a single letter is displayed every 10 s and the participant is asked if this letter was included in the previously presented list or not. This block included the option to “Retrieve List”, which redisplayed the initial set of letters but results in a loss of 10 points. The maximum number of points in a single block was 300.

Arithmetic was a task with the objective of adding two three-digit numbers together within 30 s, where the operation result was selected using “+” and “−” buttons. The maximum number of points was not specified. The score of a single block depended on the participant’s skill.

Visual monitoring was a horizontal scale displayed on the screen with a triangular pointer in the center of the scale. The pointer moved either right or left towards the edge of the scale. The reset button had to be pressed before the pointer reached the end of the scale. The closer the pointer was to the edge of the scale the higher the points rewarded for doing so but reaching the end of the scale resulted in −10 points. After the “Reset” button was clicked, the pointer returned to the center of the scale. The maximum number of points in a single block was 150.

During the auditory monitoring task, one high- or low-pitched sound was heard every 5 s. Participants were instructed to react to the high-pitched sound by pressing the button “High pitch sound”. The maximum number of points in a single block was 300.

The SynWin total score of each single block consisted of four multitasking components. In order to extract the indicator of task performance, we calculated the average total score from each of the three blocks. As the features were continuous, we kept them at their original scales.

### 2.7. Statistical Analysis

For both FFQ pro-healthy and non-healthy food products, factor analysis was performed with the method of principal components. Kaiser-Meyer-Olkin (KMO) measurement of sample adequacy and Bartlett test statistic were used to evaluate whether the obtained correlation matrix was suitable for factor analysis. Varimax rotation with Kaiser normalization was performed to extract the rotation matrix.

The relationship between factors describing healthy and non-healthy diet patterns was examined with Pearson’s correlation. Statistical significance was set at *p* < 0.05. Stepwise regression analysis was used to validate our hypothesis about dietary patterns altering the cognitive performance, where the cognitive task performance was the dependent variable and age and food consumption patterns, with their interactions, were predictors in the model. Homoscedasticity, normality of the errors, and no multicollinearity between predictors were ensured in the analysis. Because cognitive functions decline with age, the “age” factor was tested in a separate model and compared with the other predictors and their interaction terms. The next step of analysis proceeded with a backward selection method to assess the optimal combination of predictors. The main effects of predictors and interaction terms were tested and subsequently excluded if they were not significant. Then, to explain the interaction effects, we divided the participants into two groups by age (people aged less than 35 years old (*n* = 102) and above 36 years old (*n* = 79)) and conducted separate regression analyses for those subgroups.

All analyses and data preparation were conducted in the IBM SPSS Statistical Package for the Social Sciences (SPSS) version 25.

## 3. Results

### 3.1. Participants Characteristics

From 204 participants two failed to fill out all of the questionnaires and were excluded from further analyses, therefore, 202 participants were included in the study (101 males and 101 females). Participants who scored more than three standard deviations from the sample mean in BMI and all the multitasking component features from the SynWin task were excluded from further analysis.

A total of 181 participants are thus summarized in Table 1. In order to check age-related factors, we performed an Chi-squared test on contingency tables and independent sample Student *t*-test with the division of participants into age groups (people aged less than 35 years old (*n* = 102) and above 36 years old (*n* = 79)). We used 35 as a marker for age groups as Cole et al. showed cognitive decline after that age [34].

A summary of descriptive statistics is provided in Table 1. The study population is not dominated by any individual sex group, and most of the participants were employed (83.5%) and had higher education (60.8%). The age groups did not differ in sex, employment status, educational status, fatigue index (FAS), dietary knowledge, and dietary health indexes (FFQ). Some differences were noted in the health index and sleep quality during the week, which was significantly lower in the older age group (*p* < 0.05; *p* < 0.05, respectively), while BMI differences were significantly higher in the older age group (*p* < 0.001).

### 3.2. Consumption Patterns

For pro-healthy food products (KMO: 0.6; Bartlett test: chi-square: 306.4, *p* value < 0.001), the four factors together explained 63% of the cumulative variance in the extracted components. Varimax rotation with Kaiser normalization converged in six iterations. The rotated component matrix of pro-healthy food products is included in Table 2.

Component 1, which held 19% of the original variance, represented higher consumption of vegetables and fruits, with factor loadings of 0.815 and 0.850, respectively. In addition, whole grain bread also had a positive strong load in this component (0.53). Component 2 represented 16% of the original variance and was associated with fermented dairy and fresh stretched curd cheeses with factor loadings of 0.845 and 0.687. The next, component 3, held 16% of the original variance with legume vegetables (factor loading 0.854) and whole grain cereal (0.623) consumption. Component 4 represented 13% of the original variance associated with white meat (poultry and rabbit) (0.837) and fish (0.734) consumption.

Similarly, for non-healthy products (KMO: 0.7; Bartlett test: chi-square: 445.7, *p* value < 0.001) the four factors together explained 54% of the cumulative variance of the extracted components. Varimax rotation with Kaiser normalization converged in 12 iterations. The rotated component matrix of pro-healthy food products is included in Table 3.

Component 1 held 16% of the original variance associated with higher consumption of white flour baked products (0.742), fried food (0.647), butter (0.576), and lunch meat (0.631). Component 2 represented 14% of the original variance with higher consumption of carbonated soft drinks (0.774), energy drinks (0.578), alcohol (0.595), and fast food (0.631). Component 3 represented 14% of the original variance with higher consumption of animal fats such as red meat (0.603), canned meat (0.734), and lard (0.762). Component 4 held 10% of the original variance with higher consumption of refined grains (0.726) and processed semi-hard blue cheeses (0.515).

Pearson’s correlation was used to check correlations between healthy and unhealthy consumption patterns (Table 4). The fruits and vegetables component was negatively correlated with the fast food and high-sugar drinks component (*p* < 0.01), while the fermented dairy and cottages component was positively correlated with the refined grains and cheeses (*p* < 0.01) component. The component of legume vegetables and whole grain cereals was negatively correlated with the high-carbohydrates and high-fat food (HCHF food) component (*p* < 0.01) and positively correlated with the refined grains and cheeses (*p* < 0.01) component. White meat and fish frequency consumption were positively correlated with both the meat, animal fat, and the HCHF food (*p* < 0.01) components.

### 3.3. Consumption Patterns and SynWin Multitasking Performance

Linear regressions were calculated to predict the SynWin multitasking performance based on age and eight components of consumption patterns, including their interaction effects. Age was the only factor in the first step, eight components of consumption patterns were included in the second step, and the third step presented their interactions with age. The age score predictor was standardized before the analysis, while consumption patterns were components of the factor analysis.

Further analysis included the examination of single respective interactions and those effects that were found to be significant (Table S1). Effects that were not significant were subsequently excluded from the model with the lowest Akaike Information Criterion (AIC). The final model included age (β = −0.404) and the interactions of age with the HCHF food component (β = −0.15) and age with the meat and animal fat component (β = −0.161) to predict cognitive functioning performance based on the SynWin multitasking total score. The final model with the age predictor and the interaction terms was statistically significant (F(3, 177) = 15.538, *p* < 0.001) with an R^2^ of 0.208 and AIC of 1825.6 (Table 5), indicating that there was a potentially significant interaction of unhealthy consumption patterns and age with SynWin task performance.

To explain the interaction effect between age and the components of HCHF food and meat and animal fat, we divided participants into age groups (people aged less than 35 years old (*n* = 102) and above 36 years old (*n* = 79)). Next, we conducted regression analysis for these subgroups separately, with HCHF food and meat and animal fat components as predictors.

**Figure 2:**
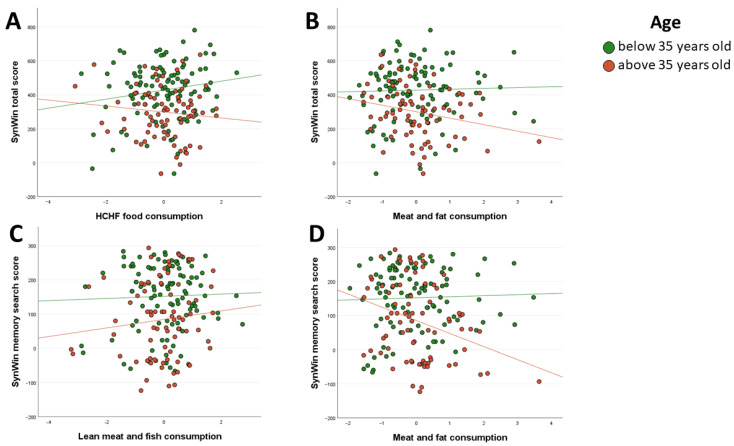
(**A**). Linear regression analyses of the associations between younger and older age groups and SynWin total score predicted by high-carbohydrates and high-fat (HCHF) consumption. (**B**). Linear regression analyses of the associations between younger and older age groups and SynWin total score predicted by meat and animal fat consumption. (**C**). Linear regression analyses of the associations between younger and older age groups and SynWin total score predicted by white meat and fish consumption. (**D**). Linear regression analyses of the associations between younger and older age groups and SynWin memory search score predicted by meat and animal fat consumption.

The models extracted from the regression analysis between the HCHF food component predictor and age groups division did not show a significant relationship between the amount of HCHF food frequency consumption and the performance of a cognitive task with age (Table 6). Examination of the interaction plot showed that as the frequency of HCHF food consumption increased in the older age group SynWin task performance decreased, while increased frequency of HCHF food consumption in the younger age group related to increased SynWin task performance (Figure 2A).

However, the model for the group above 35 years old (F(2, 76) = 2.595, *p* = 0.081) showed the pattern that higher scores of the meat and animal fat components correlated with SynWin task performance decreasing with age (β = −0.228) (Table 6). Examination of the interaction plot showed the significant effect that as the frequency of meat and animal fat consumption increased in the older age group that SynWin task performance decreased (Figure 2B). For the younger-aged group, the model implied that there is no main effect of the meat and animal fat component on SynWin task performance (Table 6).

### 3.4. Consumption Patterns and SynWin Components Performance

Linear regressions were conducted to predict SynWin components performance based on age and eight components of consumption patterns, including their interaction effects. For each SynWin component, age was the only factor in the first step, eight components of consumption patterns were included in the second step, and the third step presented their interactions with age.

Further analysis included the examination of single respective interactions and those effects that were found to be significant. Effects that were not significant were subsequently excluded from the model with the lowest AIC. The main effect of age was significant in the memory search SynWin component only (Table S2).

#### Memory Search

The final model for memory search included age (β = −0.321), the white meat and fish component (β = 0.157), the meat and animal fat component (β = −0.204), and an interaction between age and the white meat and fish (β = 0.136, however, not significant) and the meat and animal fat (β = −0.237) components. The final model was statistically significant (F(5, 175) = 7.301, *p* < 0.001) with an R^2^ of 0.173 and the lowest AIC of 1669.8 (Table 7) indicating that there was a potentially significant interaction of consumption patterns on age with the SynWin memory search task performance.

The models extracted from the analysis between various consumption patterns and the SynWin memory search score with the age groups division showed a relationship between the older age group and the performance of a memory search task with various meat patterns (F(2, 76) = 7.453, *p* < 0.001) (Table 8). The model showed that for higher scores on meat and animal fat consumption in the older age group, the SynWin memory search task score decreased (Figure 2D). Meanwhile, with higher scores on the white meat and fish component in the older age group, the SynWin memory search task score increased (Figure 2C). For the younger-aged group, the model implied that there is no main effect of various patterns of meat consumption on SynWin task performance (Table 8).

## 4. Discussion

The aim of this study was to determine whether higher consumption of specific food products might affect cognitive functioning depending on age. Our results showed that the frequency of different food consumption might moderate the effects of age on cognitive functioning in both positive and negative directions. We found a negative effect of meat and animal fat consumption on memory, especially in people of older age. Interestingly, also in the older-aged group, higher consumption of white meat and fish was related to better performance in our memory task.

Aging is associated with an increase of inflammation, which has a negative impact on synaptic plasticity and neurogenesis [42,43,44]. Saturated fat, which occurs in red meat or high-fat dairy products [45], may increase inflammation in response to hormonal changes [46]. Alternatively, omega-3 PUFA, which occurs in fish meat [47], could prevent neuroinflammation changes in the hippocampus [28].

In our study, age groups did not differ according to the means of pro-healthy and non-healthy diet indexes (Table 1). The maximum values of pro-healthy and non-healthy diet indexes were 47 and 50 respectively, which means that our respondents’ dietary patterns were neither healthy nor unhealthy. This allowed us to focus on the frequency of consumption of specific products within the same diet. In addition, 172 participants declared an omnivore diet and only 9 participants were vegetarian, but as they consumed animal protein and animal fat from food products such as eggs or cheese, we decided to include them in further analyses. However, the differences in extracted consumption pattern indexes were related to a different aspect of cognitive functioning among the age groups.

Eight factors were extracted from factor analysis, four from pro-healthy food products and four from non-healthy food products. We found three components had moderating effects of age on cognitive functioning: the HCHF food, the meat and animal fat, and the white meat and fish components. The relationships between them were checked with Pearson’s correlation (Table 2). The HCHF food component was derived from the unhealthy food products frequency consumption data and had the highest loadings of white flour baked products, fried products, lunch meat, butter, and confectionery, which typically have a high caloric content and can cause a heightened insulin response [48,49].

The meat and animal fat component was derived from the unhealthy food products frequency consumption data, but we did not interpret this component as a part of the WS diet, despite the fact that eating fried meat items is often a part of the WS diet. Because the meat and animal fat component was positively correlated with the legume vegetables and whole grain component, we interpreted it as a part of the traditional Polish cuisine. Meat and fish, lunch meat, legume vegetables, and whole grain cereal are common products in Polish cuisine. While Polish cuisine is not unhealthy it is difficult to digest. Overeating and ensuing stomach fullness with stretching of the gastric mucosa cause abnormal hormonal secretion and may worsen cognitive functioning [17,50].

The white meat and fish component was derived from healthy food products frequency consumption data. White meats have relatively low fat levels and are a good source of protein [51], while fish is rich in long-chain PUFA, which has beneficial effects on brain function [4].

Our results show that the components related to meat consumption, the meat and animal fat and the white meat and fish components, may moderate the effects of age on memory. The meat and animal fat component was negatively related to memory search performance as the main effect in the model and with interaction with age. The white meat and fish component was positively related to memory search performance as a main effect and with interaction with age (although only a tendency). After splitting the interaction effects (age × meat and animal fat and age × white meat and fish) on memory search performance into simple effects, we found that in younger individuals the model did not show a significant relation between meat components and memory search performance. However, in the older group, the model showed strong effects for both meat patterns. The meat and animal fat component showed a negative relationship with the memory search task score, with red meat and animal fat increase accompanying worse performance on the memory task. Alternatively, for the white meat and fish component, there is a positive effect of consumption frequency on the memory search task score, with more frequent consumption relating to greater memory search performance.

The role of meat, especially red meat, in brain function is still under consideration. Meat is a good source of proteins, vitamins, and minerals that the body and brain need to develop and maintain health [51,52]. For example, red meat is a good source of iron [53] and research has shown that early-life iron deficiency may have a negative impact on hippocampus-dependent memory [54]. Research has also shown that red meat and animal fats may support mental health because avoiding meat consumption was associated with depression, higher anxiety, and lower iron levels [55]. Zupo et al. (2021) also showed that lower consumption of red meat was associated with cognitive impairment and seems to have beneficial effects on cognition [56]. However, other studies showed that animal fat is also a factor contributing to the formation of inflammation in the body [57,58]. While aging is associated with an increase in inflammatory markers, it may also affect mental health [42]. Red meat is high in saturated fat and studies of high-fat diets have shown the negative effect of high saturated fat intake on the hypothalamus and the hippocampus in animals models [30,58]. In humans, higher consumption of red meat worsened attention, concentration, and information processing speed in the elderly, but has not been associated with the rate of cognitive decline [59]. It may also be related to our result, showing that with age, the frequent consumption of red meat and animal fats can decrease cognitive performance. However, there are more factors to consider with meat consumption such as gut microbiota composition and food component digestion [60].

Our results show that the components HCHF food and meat and animal fat negatively affect the performance of a cognitive task in interaction with age. However, after splitting the interaction effects (age × HCHF food and age × meat and animal fat) on cognitive task performance into simple effects, we found that in younger individuals the model did not show a significant relationship between the HCHF food index and cognitive performance. In the older group, the model showed a tendency for higher meat and animal fat consumption and worse performance in the cognitive task, but frequent eating of HCHF food did not relate to task performance as strongly.

The HCHF food component was interpreted as a WS diet-like component, due to the simple carbohydrates and fat occurring together in food products and their mutual negative effects. Consuming simple carbohydrates and fat at the same time leads the organism to higher lipoprotein levels and higher insulin levels than when consuming those organic compounds separately [61,62]. Consequently, there are disruptions in insulin signaling that may cause insulin resistance in the hippocampus and deterioration of hippocampal-dependent cognitive processes [6]. Research presented on humans has shown that one week of the WS diet weakened appetitive control, which was also negatively correlated with memory cognitive task performance [20]. Furthermore, studies on rodents also show that short-term consumption (1–7 days) of food products high in saturated fats or high in added sugar (or both) initiates inflammatory processes in the hippocampus [63]. Overall, those studies suggest that obesity is not necessarily the only problem connected with the WS diet [25], it seems that it could also lead to cognitive impairments due to changes in digestive signaling pathways.

Our results showed that changes in cognitive performance may occur along with changes in the frequency of food products consumption, especially in the case of memory. However, the estimation of cognitive performance was not assessed at baseline, which is the main limitation of the present study. It cannot be confidently stated that those nutritional choices caused changes in the performance of the cognitive task, although independent mean comparison tests based on the two age groups did not show any essential differences between the groups.

The age groups did not differ in the fatigue score or lifestyle factors, such as education, employment, physical activity, or smoking. In addition, they did not differ in knowledge about nutrition and on the main dietary metrics, the pro-healthy diet index and non-healthy diet index. However, the age groups differed in the sleep quality factor on weekdays. Young adults were more likely to sleep irregularly than midlife adults, but on weekends those differences were not observed. The age groups also differed in terms of BMI and health factors, which may also be reflected in cognitive functioning. Younger adults have lower BMI scores and rate their health better than midlife adults. Considering that we examined physically and mentally healthy people, the observed cognitive performance may depend on the frequency of consumption of food products. Research also showed that cognitive performance may be affected by stress [64]. Although FAS includes questions about mental fatigue, which is a symptom of stress [37], the stress was not examined in our study. In future research, a stress questionnaire should be included in the study to eliminate this factor as well.

## 5. Conclusions

Our results showed that the frequency of different food consumption could be seen as an important factor modifying age-related cognitive decline. One of the more significant findings from this study is that the type of meat consumed, white or red, relates differently to memory performance in the group of older participants. Eating more white meat and fish was related to better memory in the older group and, conversely, consuming more red meat and animal fat was accompanied by lower scores on the memory task. Those findings are especially interesting in light of the recently published paper by Noble et al. (2021) on the impact of an unhealthy diet on rat memory performance and the hippocampus [65]. The results in our research support the idea that diet may be a protective (or worsening) factor in age-related cognitive decline.

## Figures and Tables

**Table 1:** Participant characteristics, descriptive statistics, and test differences for: I. all participants, II. each age group.

Characteristics	I. All (*n* = 181) Number/Mean (SD)	II. Age Group ≤ 35 (*n* = 102) Number/Mean (SD)	II. Age Group ≥ 36 (*n* = 79) Number/Mean (SD)	II. Test Differences for Age Groups
Sex	♂ 92; ♀ 89	♂ 46; ♀ 56	♂ 46; ♀ 33	X^2^: 3.071
Age	35.5 (9.2)	28.7 (4.3)	44.2 (5.8)	t: −20.061 ***
Employment	83.50%	82.4%	84.8%	X^2^: 0.194
Education				X^2^: 0.858
secondary	38.1%	35.3%	41.8%	
vocational	1.1%	1%	1.3%	
higher	60.8%	63.7%	57%	
Health	7.2 (1.7)	7.5 (1.6)	6.9 (1.7)	t: 2.235 *
BMI	24.7 (4.9)	23.1 (4.0)	26.9 (5.2)	t: −5.534 ***
FAS score	22.83 (7.3)	23.1 (7.3)	22.4 (7.2)	t: 0.638
Dietary knowledge	12.4 (4.5)	11.9 (4.4)	13.0 (4.5)	t: −1.660
Pro-Healthy Diet Index	20.1 (10.0)	20.0 (10.5)	20.2 (9.4)	t: −0.140
Non-Healthy Diet Index	16.3 (8.2)	16.9 (8.7)	15.6 (7.5)	t: 1.08
Smoking	22.7%	19.6%	26.6%	X^2^: 1.236
Sleeping quality (weeks):				X^2^: 5.919 *
7–8 h	61.9%	69.6%	51.9%	
<7 h or >8 h	38.1%	30.4%	48.1%	
Sleep quality (weekends):				X^2^: 0.297
7–8 h	63.0%	64.7%	60.8%	
<7 h or >8 h	37.0%	35.3%	39.2%	
Physical activity				X^2^:2.792
sedentary or light	59.1%	63.7%	53.2%	
medium active	37.6%	32.4%	44.3%	
vigorously active	3.3%	3.9%	2.5%	
Diet type				X^2^: 0.002
omnivore	95%	95.1%	94.9%	
vegan	5%	4.9%	5.1%	

SD = standard deviation; BMI = Body Mass Index; FAS = Fatigue Assessment Scale; * *p* value < 0.05, *** *p* value < 0.001.

**Table 2:** Rotated component matrix of pro-healthy food products.

	Fruit and Vegetables	Fermented Dairy, Cottages	Legumes, Whole Grain	White Meat and Fish
whole meal bread	0.536	0.073	0.155	0.017
whole grain cereal	0.163	0.373	0.623	−0.046
milk	0.387	0.456	−0.185	0.208
fermented dairy	0.091	0.845	0.02	0.005
fresh stretched curd cheeses	0.016	0.687	0.404	0.12
white meat	0.040	0.182	−0.130	0.837
fish	0.072	−0.061	0.443	0.734
legume vegetables	0.128	−0.038	0.854	0.099
fruits	0.850	0.083	0.037	0.048
vegetables	0.815	0.052	0.095	0.025

Varimax rotation with Kaiser normalization converged in six iterations.

**Table 3:** Rotated component matrix of non-healthy food products.

	High-Carbohydrates, High-Fat Food (HCHF)	Fast Food, High-Sugar Drinks	Meat and Animal Fat	Refined Grains, Cheeses;
white flour baked products	0.742	0.060	−0.190	0.076
refined grains	0.043	0.057	−0.014	0.726
fast food	0.022	0.631	0.265	0.282
fried food	0.647	0.235	0.025	0.167
butter	0.576	−0.202	0.116	0.172
lard	−0.049	0.073	0.762	0.281
moldy, processed, semi-hard cheeses	0.318	−0.029	0.145	0.515
lunch meat	0.631	−0.033	0.343	−0.170
red meat	0.395	−0.008	0.603	−0.187
confectionery	0.434	0.350	−0.158	0.285
canned meat	−0.002	0.295	0.734	−0.022
carbonated soft drinks	0.143	0.774	0.054	−0.054
energy drinks	−0.279	0.578	0.451	0.029
alcohol	0.026	0.595	0.039	−0.479

Varimax rotation with Kaiser normalization converged in 12 iterations.

**Table 4:** Pearson correlations among healthy and non-healthy factors describing food consumption patterns from the FFQ questionnaire.

	High-Carbohydrates, High-Fat Food	Fast Food, High-Sugar Drinks	Meat and Animal Fat	Refined Grains, Cheeses
Fruit and vegetables;	0.06	−0.193 **	−0.08	0.101
Fermented dairy, cottages;	0.105	−0.091	−0.041	0.203 **
Legume vegetables, whole grain	−0.315 **	0.015	0.306 **	0.218 **
White meat and fish	0.283 **	0.073	0.379 **	−0.119

** *p* value < 0.01.

**Table 5:** Summary of linear stepwise regression analysis for various consumption patterns predicting SynWin multitasking performance in the group of all participants (*n* = 181).

**Stepwise Regression on SynWin Multitasking Performance**									
**Variables**	**B**	**SE**	**β**	**t**	***p***	**R^2^**	**ΔR^2^**	**F Statistic**	**AIC**
Step 1					0.000	0.155	-	32.839	1833.5
Age	−67.087	11.707	−0.394	−5.731	0.000				
Step 2					0.457	0.162	0.007	0.787	1835.9
Age	−66.730	11.736	−0.392	−5.686	0.000				
HCHF food	10.498	11.722	0.062	0.896	0.372				
Meat and animal fat	−10.308	11.735	−0.060	−0.878	0.381				
Step 3					0.003	0.217	0.054	6.065	1827.7
Age	−68.677	11.461	−0.403	−5.992	0.000				
HCHF food	9.44	11.512	0.055	0.82	0.413				
Meat and animal fat	−12.668	11.52	−0.074	−1.100	0.273				
Age × HCHF food	−24.734	12.209	−0.138	−2.026	0.044				
Age × Meat and animal fat	−34.386	13.442	−0.175	−2.558	0.011				
**Final Model on SynWin Multitasking Performance**									
**Variables**	**B**	**SE**	**β**	**t**	***p***	**R^2^**	**ΔR^2^**	**F Statistic**	**AIC**
Final model					0.000	0.208	-	15.538	1825.6
Age	−68.913	11.446	−0.404	−6.021	0.000				
Age × HCHF food	−26.852	12.081	−0.150	−2.223	0.028				
Age × Meat and animal fat	−31.740	13.293	−0.161	−2.388	0.018				

ΔR^2^ = difference in the proportion of variance explained in reference to the step 1 regression; B = unstandardized regression coefficient; SE = standard error; β = standardized regression coefficient; AIC = Akaike Information Criterion. The dependent variable was the total SynWin score. HCHF = High-carbohydrates and high-fat.

**Table 6:** Explanations of the interaction effects (age × HCHF food and age × meat and fat age) showing a simple effect for younger and older age groups on SynWin multitasking performance predicted by the frequency of HCHF food consumption and meat and animal fat frequency consumption.

Variables	B	SE	β	t	*p*	R^2^	F Statistic
Age below 35					0.22	0.03	1.535
HCHF food	26.589	15.441	0.170	1.722	0.088		
Meat and animal fat	5.624	15.308	0.036	0.367	0.714		
Age above 35					0.081	0.064	2.595
HCHF food	−16.063	17.892	−0.100	−0.898	0.372		
Meat and animal fat	−37.382	18.187	−0.228	−2.055	0.043		

B = unstandardized regression coefficient; SE = standard error; β = standardized regression coefficient. HCHF = High-carbohydrates and high-fat. The dependent variable was the total SynWin score.

**Table 7:** Summary of linear stepwise regression analysis for various consumption patterns predicting the impact on SynWin memory search performance for all participants (*n* = 181).

**Stepwise Regression on SynWin Memory Search Score**									
**Variables**	**B**	**SE**	**β**	**t**	***p***	**R^2^**	**ΔR^2^**	**F Statistic**	**AIC**
Step 1					0.000	0.102	-	20.229	1676.7
Age	−34.153	7.593	−0.319	−4.498	0.000				
Step 2					0.125	0.130	0.029	1.939	1676.9
Age	−33.040	7.557	−0.308	−4.372	0.000				
White meat and fish	12.411	8.565	0.116	1.449	0.149				
HCHF food	4.671	7.916	0.044	0.590	0.556				
Meat and animal fat	−16.743	8.220	−0.156	−2.037	0.043				
Step 3					0.007	0.189	0.058	4.152	1670.3
Age	−34.215	7.389	−0.319	−4.630	0.000				
White meat and fish	14.273	8.387	0.133	1.702	0.091				
HCHF food	4.948	7.764	0.046	0.637	0.525				
Meat and animal fat	−20.356	8.146	−0.190	−2.499	0.013				
Age × White meat and fish	16.551	7.683	0.171	2.154	0.033				
Age × HCHF food									
Age × Meat and animal fat	−13.538	8.095	−0.120	−1.672	0.096				
	−29.585	9.676	−0.239	−3.058	0.003				
**Final Model on SynWin Memory Search Score**									
**Variables**	**B**	**SE**	**β**	**t**	***p***	**R^2^**	**ΔR^2^**	**F Statistic**	**AIC**
Final model					0.000	0.173	-	7.301	1669.8
Age	−34.399	7.412	−0.321	−4.641	0.000				
White meat and fish	16.831	8.031	0.157	2.096	0.038				
Meat and animal fat	−21.854	8.117	−0.204	−2.692	0.008				
Age × White meat and fish	13.217	7.491	0.136	1.765	0.079				
Age × Meat and animal fat	−29.290	9.699	−0.237	−3.020	0.003				

ΔR^2^ = difference in the proportion of variance explained; B = unstandardized regression coefficient; SE = standard error; β = standardized regression coefficient; AIC = Akaike Information Criterion. The dependent variable was the memory search SynWin score. HCHF = High-carbohydrates and high-fat.

**Table 8:** Explanations of the interaction effects (age × white meat and fish and age × meat and animal fat) showing a simple effect for younger and older age groups on SynWin memory search score predicted by the frequency of white meat and fish consumption and meat and animal fat frequency consumption.

Variables	B	SE	β	t	*p*	R^2^	F Statistic
Age below 35					0.913	0.002	0.091
White meat and fish	2.322	9.738	0.026	0.238	0.812		
Meat and animal fat	2.281	9.360	0.026	0.244	0.808		
Age above 35					0.001	0.164	7.453
White meat and fish	32.732	13.303	0.283	2.461	0.016		
Meat and animal fat	−52.614	14.135	−0.428	−3.722	0.000		

B = unstandardized regression coefficient; SE = standard error; β = standardized regression coefficient. The dependent variable was the SynWin memory search score.

## Data Availability

Data available upon request.

## Supplementary Materials

The following are available online at https://www.mdpi.com/article/10.3390/nu13082504/s1, Table S1: Summary of linear stepwise regression analysis for various consumption patterns predicting SynWin multitasking performance in the group of all participants (*n* = 181), Table S2: Summary of linear stepwise regression analysis for various consumption patterns predicting SynWin memory search performance in the group of all participants (*n* = 181).
