# Supplementary material for: Age-Related Cognitive Decline May Be Moderated by Frequency of Specific Food Products Consumption
Source: Nutrients. 2021 Jul 22;13(8):2504. doi: 10.3390/nu13082504 (PMC8399560; doi:10.3390/nu13082504)
Supplement: Supplementary file 1 [file nutrients-13-02504-s001.zip › nutrients-1266532-supplementary.pdf]

Table S1. Summary of linear stepwise regression analysis for various consumption patterns predicting SynWin multitasking performance in the group of all participants (N = 181).

Stepwise regression on SynWin multitasking performance									
**Variables**	**B**	**SE**	**β**	**t**	**p**	**R^2**	**ΔR^2**	**F statistic**	**AIC**
**Step 1**					0.000	0.155	-	32.839	1833.5
Age	-67.087	11.707	-0.394	-5.731	0.000				
**Step 2**					0.498	0.19	0.035	0.924	1841.8
Age	-69.166	12.916	-0.406	-5.355	0.000				
Fruit and vegetables	-14.806	12.252	-0.087	-1.209	0.229				
Fermented dairy	-3.401	12.179	-0.020	-0.279	0.780				
Legume veg. and wholegrain	-2.633	13.605	-0.015	-0.194	0.847				
Lean meat and fish	17.293	13.581	0.101	1.273	0.205				
HCHF food	6.058	13.148	0.036	0.461	0.646				
Fast food and sugary drinks	-16.958	12.108	-0.100	-1.401	0.163				
Meat and fat	-17.266	13.548	-0.101	-1.274	0.204				
Refined grains, cheeses	-10.143	13.577	-0.060	-0.747	0.456				
**Step 3**					0.062	0.259	0.069	1.909	1841.6
Age	-69.585	12.928	-0.408	-5.382	0.000				
Fruit and vegetables	-13.417	12.491	-0.079	-1.074	0.284				
Fermented dairy	-15.894	12.495	-0.093	-1.272	0.205				
Legume veg. and wholegrain	-4.674	13.838	-0.027	-0.338	0.736				
Lean meat and fish	19.394	13.696	0.114	1.416	0.159				
HCHF food	6.787	13.139	0.040	0.517	0.606				
Fast food and sugary drinks	-17.590	12.099	-0.103	-1.454	0.148				
Meat and fat	-21.204	13.932	-0.124	-1.522	0.130				
Refined grains, cheeses	-1.244	13.985	-0.007	-0.089	0.929				
Age x Fruit and vegetables	-0.853	13.909	-0.005	-0.061	0.951				
Age x Fermented dairy	-1.205	12.448	-0.007	-0.097	0.923				
Age x Legume veg. and wholegrain	-3.527	14.747	-0.019	-0.239	0.811				
Age x Lean meat and fish	12.370	12.793	0.080	0.967	0.335				
Age x HCHF food	-31.180	14.328	-0.174	-2.176	0.031				
Age x Fast food and sugary drinks	-9.248	14.205	-0.048	-0.651	0.516				
Age x Meat and fat	-47.800	16.608	-0.243	-2.878	0.005				
Age x Refined grains, cheeses	-13.561	12.960	-0.076	-1.046	0.297				

[1]ΔR^2 = difference in the proportion of variance explained in reference to the step 1 regression; β = standardized regression coefficient. The dependent variable was the total SynWin score.

Table S2. Summary of linear stepwise regression analysis for various consumption patterns predicting SynWin memory search performance in the group of all participants (N = 181).

Stepwise regression on SynWin multitasking performance									
**Variables**	**B**	**SE**	**β**	**t**	**p**	**R^2**	**ΔR^2**	**F statistic**	**AIC**
**Step 1**					0.000	0.102	-	20.229	1676.7
Age	-34.153	7.593	-0.319	-4.498	0.000				
**Step 2**					0.366	0.145	0.044	1.099	1683.7
Age	-36.114	8.345	-0.337	-4.328	0.000				
Fruit and vegetables	-2.551	7.916	-0.024	-0.322	0.748				
Fermented dairy	1.518	7.869	0.014	0.193	0.847				
Legume veg. and wholegrain	-6.460	8.790	-0.060	-0.735	0.463				
Lean meat and fish	11.400	8.775	0.106	1.299	0.196				
HCHF food	2.960	8.495	0.028	0.348	0.728				
Fast food and sugary drinks	-6.996	7.823	-0.065	-0.894	0.372				
Meat and fat	-14.375	8.753	-0.134	-1.642	0.102				
Refined grains, cheeses	-8.535	8.772	-0.080	-0.973	0.332				
**Step 3**					0.063	0.218	0.073	1.901	1683.5
Age	-36.137	8.355	-0.337	-4.325	0.000				
Fruit and vegetables	-3.544	8.072	-0.033	-0.439	0.661				
Fermented dairy	-5.672	8.074	-0.053	-0.703	0.483				
Legume veg. and wholegrain	-8.489	8.943	-0.079	-0.949	0.344				
Lean meat and fish	13.720	8.850	0.128	1.550	0.123				
HCHF food	3.294	8.491	0.031	0.388	0.699				
Fast food and sugary drinks	-8.318	7.819	-0.078	-1.064	0.289				
Meat and fat	-18.678	9.003	-0.174	-2.075	0.040				
Refined grains, cheeses	-2.560	9.038	-0.024	-0.283	0.777				
Age x Fruit and vegetables	-0.415	8.989	-0.004	-0.046	0.963				
Age x Fermented dairy	-6.453	8.044	-0.060	-0.802	0.424				
Age x Legume veg. and wholegrain	-9.662	9.530	-0.082	-1.014	0.312				
Age x Lean meat and fish	15.263	8.267	0.158	1.846	0.067				
Age x HCHF food	-18.414	9.259	-0.163	-1.989	0.048				
Age x Fast food and sugary drinks	-6.793	9.180	-0.057	-0.740	0.460				
Age x Meat and fat	-31.564	10.733	-0.255	-2.941	0.004				
Age x Refined grains, cheeses	-8.496	8.375	-0.075	-1.014	0.312				

[1]ΔR^2 = difference in the proportion of variance explained; β = standardized regression coefficient. The dependent variable was the memory search SynWin score.
